# Emerging Synthetic Bioluminescent Reactions for Non-Invasive Imaging of Freely Moving Animals

**DOI:** 10.3390/ijms25137338

**Published:** 2024-07-04

**Authors:** Takahiro Kuchimaru

**Affiliations:** Center for Molecular Medicine, Jichi Medical University, Tochigi 329-0498, Japan; kuchimaru@jichi.ac.jp

**Keywords:** synthetic bioluminescent reaction, bioluminescence imaging, freely moving animals, near-infrared bioluminescence, firefly luciferase, NanoLuc

## Abstract

Bioluminescence imaging (BLI) is an indispensable technique for visualizing the dynamics of diverse biological processes in mammalian animal models, including cancer, viral infections, and immune responses. However, a critical scientific challenge remains: non-invasively visualizing homeostatic and disease mechanisms in freely moving animals to understand the molecular basis of exercises, social behavior, and other phenomena. Classical BLI relies on prolonged camera exposure to accumulate the limited number of photons that traveled from deep tissues in anesthetized or constrained animals. Recent advancements in synthetic bioluminescence reactions, utilizing artificial luciferin–luciferase pairs, have considerably increased the number of detectable photons from deep tissues, facilitating high-speed BLI to capture moving objects. In this review, I provide an overview of emerging synthetic bioluminescence reactions that enable the non-invasive imaging of freely moving animals. This approach holds the potential to uncover unique physiological processes that are inaccessible with current methodologies.

## 1. Introduction

**Figure 1:**
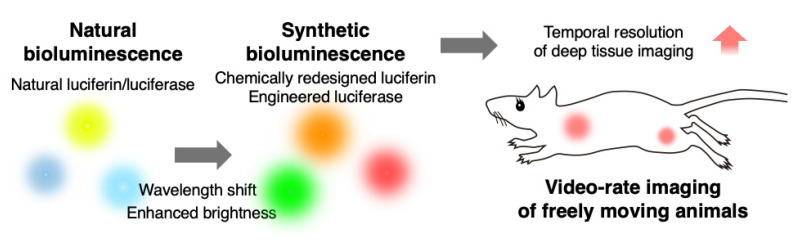
The potential of synthetic bioluminescent reactions for imaging freely moving animals.

The direct visualization of cellular behavior and molecular activities provides strong evidence for our understanding of living systems [1,2,3,4,5,6]. The discovery of the green fluorescent protein transformed our approach to optical imaging by allowing the genetic incorporation of light-emitting agents into cells and animals [7]. Currently, a variety of genetically encoded light-emitting agents are available for optical imaging, falling into two major categories: fluorescence imaging and bioluminescence imaging. Fluorescence imaging utilizes light-emitting agents (e.g., fluorescent proteins) that require excitation light to generate fluorescence signals. In contrast, bioluminescence imaging (BLI) relies on the light signals generated by the enzymatic reactions between luciferins and luciferases. For example, the reaction between d-luciferin and firefly luciferase (Fluc) is a widespread bioluminescence system in life science research. Fluc catalyzes the oxidation of its natural substrate, d-luciferin, with adenosine triphosphate (ATP) and Mg^2+^ as co-factors, resulting in the emission of yellow-green photons with a peak emission wavelength of 565 nm (λmax = 565 nm) [8,9,10]. To utilize bioluminescent reactions in living cells and animals, luciferins are externally administered and delivered to luciferases. This system offers substantial benefits over fluorescence imaging, particularly in the imaging of living animals. Light is strongly scattered and absorbed by living tissues, hindering its penetration into deep tissues [11]. Fluorescence imaging requires a two-way path in living tissues to complete its imaging: excitation light must reach the target, and fluorescent light must reach the body surface. In addition, living tissues contain abundant fluorescent molecules, such as vitamins and lipids [12,13]. These autofluorescence signals are not negligible in bulky tissue imaging, and significantly reduce the signal-to-background level by elevating background signals. In contrast, BLI benefits from the single-path travel of photons and the absence of autoluminescence signals from living tissues. This allows for a detection sensitivity that is 100–1000 times greater than that of fluorescence imaging [14]. However, the photon output in bioluminescence reactions is typically 10–100 times lower than that in fluorescence imaging. This necessitates the prolonged exposure (seconds to minutes) of the subjects to photodetectors to accumulate sufficient photon signals from targets within deep tissues. Consequently, classical BLI techniques lack temporal resolution, and are unsuitable for the imaging of moving objects at video rates, which typically require over 30 image acquisitions per second Although improved non-invasive measurements are highly desirable in various biomedical fields, such as exercise science and social behavior studies involving free moving animals, current BLI methods fall short of meeting these demands. To address this issue, ongoing efforts in the field have demonstrated the fact that synthetic bioluminescence reactions can generate sufficient photons for the video-rate imaging of targets within deep tissues. In this review, I discuss emerging synthetic bioluminescent reactions that offer improved tissue penetration and photon outputs over classical bioluminescence reactions, making them suitable for the non-invasive imaging of freely moving animals (Figure 1).

## 2. Bioluminescence Imaging of Freely Moving Animals

The non-invasive tracking of mammalian cells in mice was firstly demonstrated in 1998 using a natural firefly bioluminescent reaction with the pair of d-luciferin and Fluc [15]. A key advantage of this system is the high biocompatibility of d-luciferin due to its high water solubility, stability in blood circulation, and relatively ubiquitous diffusion in animal tissues. Subsequently, marine luciferases, such as Renilla luciferase (Rluc), have been engineered as another option for reporter genes for BLI in small animals [16]. Unlike the firefly bioluminescent reaction, Rluc promotes the bioluminescence emission by catalyzing the oxidation of coelenterazine. Even though Fluc and Rluc were widely adopted for imaging anesthetized animals [17,18,19], increased attention has been directed toward aequorin, one of the authentic bioluminescent systems, for imaging freely moving animals. 

Aequorin, isolated from the hydromedusa *Aequorea* by Shimomura et al. in 1962 [20], offers a unique mechanism for generating bioluminescence. Aequorin catalyzes the oxidation of coelenterazine in a Ca^2+^ concentration-dependent manner [21,22]. Neuroscientists naturally paid attention to the Ca^2+^-dependent bioluminescent activity of aequorin to monitor neuronal firing. The first time, Baubet et al. were the first to demonstrate aequorin’s potential as a genetic indicator to report neuronal firing via voltage-dependent Ca^2+^ influx in primary culture neurons [23]. This opened the way for monitoring neuronal activation in freely moving animals, including zebra fish and fruit fly [24,25,26]. However, the applications of aequorin-based bioluminescent systems are restricted to small fishes and insects because of the limited photon delivery in bulky tissues owing to the modest bioluminescent output and less tissue penetration of its visible light spectrum, even though an engineered aequorin fused with a fluorescent protein successfully exhibited a red-shifted wavelength spectrum [27,28,29]. 

In contrast, firefly bioluminescent systems are better suited for BLI in small rodents. Indeed, Hamada et al. elegantly demonstrated that the circadian rhythm could be continuously monitored in the olfactory bulb and skin of freely moving mice expressing clock gene reporters using the firefly bioluminescent system [30]. Following this study, several other studies have employed firefly bioluminescent systems for the long-term monitoring of circadian rhythms in freely moving mice [31,32]. Despite these successful studies, the application of BLI with natural firefly bioluminescent reactions is limited to imaging superficial targets in freely moving mice. To access deeper organs in rodents, natural firefly bioluminescent reactions are restricted by the modest photon output and limited tissue penetration of visible light.

## 3. Near-Infrared Firefly Bioluminescent Reactions

**Figure 2:**
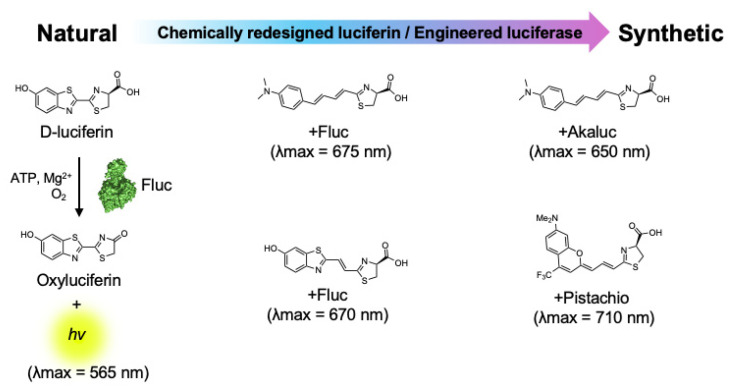
Synthetic bioluminescent reactions based on the firefly bioluminescent system.

The d-luciferin/Fluc reaction remains the gold standard for the non-invasive imaging of small animals [33]. Although it emits yellow-green bioluminescence with a relatively longer wavelength than the blue-green bioluminescence generated by marine luciferin–luciferase reactions, the tissue penetration of light gradually improves for wavelengths longer than 600 nm [11,34]. In nature, bioluminescence with the most red-shifted emission (λmax = 628 nm) has been found in the *Phrixothrix* railroad-worm, which utilizes the d-luciferin and *Phrixothrix* luciferase reaction [35]. Also, the mammalian-optimized North American firefly luciferase (luc2) displays a red-sifted emission (λmax = 609 nm) at 37 °C [36]. However, natural bioluminescent reactions that emit light with a peak wavelength exceeding 630 nm have not yet been discovered. Meanwhile, the near-infrared (NIR) wavelength (>650 nm) maximizes the photon transmission in biological tissues because NIR photons experience reduced absorption and scattering [34]. This has encouraged the exploration of synthetic bioluminescent reactions that generate NIR bioluminescence via the chemical redesign of luciferins and the protein engineering of luciferases (Figure 2). 

To achieve NIR bioluminescence using firefly luciferase, Iwano et al. identified AkaLumine, a d-luciferin analogue, with a simple aromatic structure, and extended π-conjugation, which generates NIR bioluminescence (λmax = 675 nm) in reaction with Fluc [37]. Another early approach utilized bioluminescence resonance energy transfer (BRET) to achieve a bioluminescence shift into an NIR wavelength spectrum. Kojima et al. incorporated an NIR fluorophore as a BRET donor in aminoluciferin, enabling the precise tuning of bioluminescence wavelengths ranging from 650 nm to 800 nm in reaction with Fluc [38,39]. Following these works, Jathoul et al. identified infraluciferin (iLH2) as a Fluc substrate that generates NIR-shifted bioluminescence (λmax = 670 nm) [40]. Recently, several attempts have been made to identify luciferin analogues that generate NIR bioluminescence in reactions with Fluc [41,42]. Kamiya et al. achieved the most NIR-shifted bioluminescent reaction by further extending the π-conjugation of AkaLumine, resulting in a bioluminescence emission peak at 765 nm upon the reaction with Fluc [43]. Additionally, Hall et al. proposed an elegant strategy using click beetle luciferases that exhibit wavelengths exceeding 740 nm when reacted with hydroxy-naphtha[2,1] thiazole luciferins [44]. Similarly, Love et al. developed unique synthetic NIR bioluminescent reactions (λmax = 710 nm) by combining trifluoromethyl coumarin luciferins and an engineered Fluc [45]. Although these attempts have yielded remarkable insights into the chemical redesign of bioluminescent reactions for NIR BLI and showcased their potential for deep-tissue imaging, most of the reactions currently exhibit photon outputs that are one or two orders of magnitude lower than that of the d-luciferin/Fluc reaction. To address this challenge, Iwano et al. employed a direct evolution strategy on Fluc to create Akaluc, which has a high catalytic activity for AkaLumine [46] (Table 1).

The AkaLumine/Akaluc system offers a robust NIR-shifted bioluminescence wavelength (λmax = 650 nm), and greatly increases the bioluminescence output. Consequently, the AkaLumine/Akaluc bioluminescent reaction exceptionally surpasses the detection sensitivity for deep tissue targets over BLI using the d-luciferin/Fluc reaction. The AkaLumine/Akaluc bioluminescence imaging (AkaBLI) improved the detection sensitivity of targets, achieving a 50-fold increase in the lungs and a 1500-fold increase in the brain as compared to the d-luciferin/Fluc reaction. The enhanced sensitivity in brain imaging is attributed to the combination of NIR bioluminescent properties and improved permeability of AkaLumine across the blood–brain barrier (BBB). The AkaBLI enables the detection of a small population of neurons in the intact brains of freely moving mice and marmosets. Although AkaBLI is eligible for the non-invasive imaging of freely moving animals, it requires some improvements. Specifically, the high hydrophobicity of AkaLumine necessitates its preparation in an acidic aqueous solution [60], which may cause toxicity during repeated administration for the long-term monitoring of freely moving animals. In addition, AkaLumine emits slight autoluminescence in the liver, reducing the signal-to-noise ratio in BLI [59]. Although the exact mechanism of autoluminescence is not fully understood, it is likely related to the hydrophobicity of AkaLumine, leading to the accumulation of AkaLumine in the liver and potentially intensifying detectable autoluminescence. Optimizing the physicochemical properties of AkaLumine would address these limitations, enhancing its suitability for imaging freely moving animals.

## 4. Bright Marine Bioluminescent Reactions

**Figure 3:**
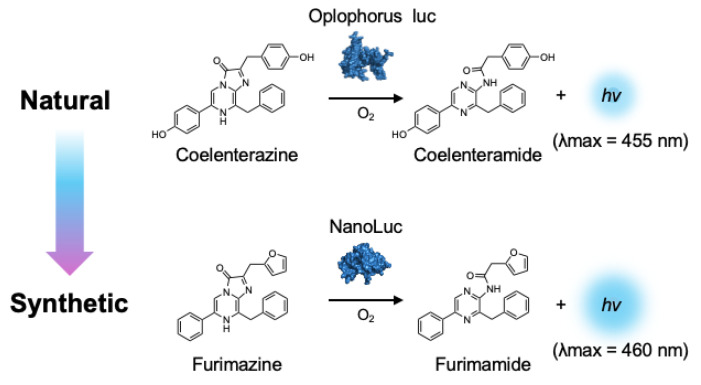
A synthetic bioluminescent reaction based on the bioluminescent system of the deep sea shrimp.

In addition to extending the bioluminescence wavelength into the NIR region, an alternative strategy involves enhancing the bioluminescence output to overcome tissue absorption and scattering. To this aim, NanoLuc-based bioluminescent systems represent a powerful technology. NanoLuc is an engineered luciferase that is derived from the Oplophorus luciferase [61,62]. In 2000, Inoue et al. isolated the Oplophorus luciferase from the deep sea shrimp *Oplophorus gracilirostris* and characterized its bioluminescent reaction [63,64]. Subsequently, Hall et al. elegantly identified furimazine, a highly optimized luciferin analogue designed for NanoLuc, as a replacement for its natural substrate, coelenterazine [61] (Figure 3). The synthetic bioluminescent reaction with frurimazine and NanoLuc displayed exceptional bioluminescent output due to its rapid catalytic reaction rate, 43 times faster than that of the d-luciferin/Fluc reaction [63]. A recent study elucidated the exact interaction mechanisms between furimazine and NanoLuc, and found that random mutagenesis in NanoLuc did not lead to a significant increase in its catalytic activity on furimazine [65]. This implicates that the straightforward direct evolution of NanoLuc is not a viable strategy for the development of brighter bioluminescent reactions, whereas BRET-based strategies have successfully enhanced the bioluminescence outputs of NanoLuc. Suzuki et al. engineered NanoLuc fused with mTrquoise2 or mNeonGreen to develop CeNL and GeNL, respectively [66]. This attempt benefited from the high quantum yield of the fluorescent proteins, resulting in an approximately a 2-fold increase in the bioluminescence output compared to NanoLuc alone [66].

Despite these remarkable accomplishments, optimizing NanoLuc-based bioluminescent systems for deep-tissue imaging requires key improvements in bioluminescence wavelength and the aqueous solubility of the substrate. The blue photons generated by the furimazine/NanoLuc reaction are highly absorbed and scattered within the living tissues. Thus, red-shifted NanoLuc-based systems are anticipated to offer efficient bioluminescence transmission from deep tissues to the body surface. Consequently, several research groups have successfully developed engineered luciferases including ReNano-lantern, Antares and LumiScarlet. These systems generate bioluminescence with peak emissions in the 560–580 nm wavelength via BRET by fusing NanoLuc with orange-red fluorescent proteins [67,68,69]. Furthermore, the limited aqueous solubility of furimazine restricts the delivery of sufficient substrates in animals, thereby impeding the maximization of the luciferin–luciferase reaction in living tissues. Su et al. demonstrated that furimazine analogues, hydro-furimazine and fluorofurimazine, display the high aqueous solubility and enhanced bioluminescent outputs in the BLI of mice by facilitating improved substrate delivery to target tissues [70]. Notably, another synthetic substrate, cephalofurimazine, further improved delivery to the brain across BBB, and the enhanced bioluminescence output was sufficient for monitoring freely moving mice [71].

Taken together, although the past decade has seen considerable advancements in establishing methodologies for small animal imaging using the NanoLuc-based bioluminescent systems, future challenges include achieving a further shift in wavelength into the NIR region to maximize detection sensitivity for deep-tissue imaging in small animals.

## 5. Bioluminescence Indicators

In addition to the bioluminescence reactions suited for freely moving animals, a crucial goal is to develop molecular imaging methods using bioluminescence indicators. In an early study, Inagaki et al. developed a Nanolantern-based voltage indicator and demonstrated the monitoring of neuronal activity in the brain of freely moving mice; however, this required the surgical removal of the skull [72]. Recently, Wu et al. successfully monitored the kinase activity in the intact brain tissue using a NanoLuc-based kinase indicator [73]. These studies lay promising foundations for the non-invasive molecular imaging of freely moving mice in combination with optimized synthetic bioluminescent reactions. Meanwhile, as compared to these NanoLuc-based methods, there have been fewer studies to design genetic indicators using firefly bioluminescent reactions for small animal imaging. This is primarily due to the large molecular size and structural complexity of firefly luciferases, which hinder protein engineering efforts. Historically, genetic indicators with firefly bioluminescent reactions have employed the split-protein reconstitution strategy, originally developed for fluorescent protein indicators [74,75,76,77,78,79]. However, unlike fluorescence proteins, the reconstituted firefly luciferase does not fully regain its catalytic activity, resulting in significantly diminished bioluminescent outputs [80]. This would be a critical limitation of the current indicator design of firefly luciferases for wide-rate animal imaging. Establishing design methodologies for bright NIR firefly bioluminescent indicators holds immense potential for the non-invasive molecular imaging of freely moving animals.

## 6. Outlook and Conclusions

Over the past decade, efforts in engineering natural bioluminescent reactions have led to various approaches for imaging freely moving animals. The coming decade is expected to see further refinements of synthetic bioluminescent reactions, expanding our options for small animal imaging. Although the development of highly tissue-penetrable and bright bioluminescence tools is a central focus of this review, improving related areas would refine the imaging of freely moving animals. For instance, optical systems with an electron-multiplying charge-coupled device have been adopted to achieve high temporal resolution and sensitive detection in the bioluminescence imaging of freely moving animals. However, they lack spatial resolution in wide-field imaging because of scalability constraints on the photon sensor. To address this issue, a complementary metal-oxide-semiconductor camera with ultra-low background noise holds promise for constructing spatially expandable imaging systems. Luciferin supplementation is another critical factor for long-term animal observation. A single injection of luciferins can sustain imaging for an hour, and an implantable pump system can continuously supply the luciferin for several hours or even days. However, the ideal scenario would involve natural and noninvasive luciferin delivery through food and water, eliminating the need for procedures that could impact animal behavior. Because of the limited uptake of luciferins via this route, luciferin analogues with higher Michaelis constants, which can saturate luciferin–luciferase reactions at lower tissue concentrations, are needed. By integrating these approaches, more reliable and scalable methodologies for imaging freely moving animals can be developed.

## Figures and Tables

**Table 1:** The luciferin–luciferase reactions generating NIR bioluminescence.

Luciferin	Luciferase	λmax	Reference
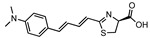 AkaLumine	Fluc Akaluc	675 650	[37,46,47,48,49,50,51,52,53,54,55,56]
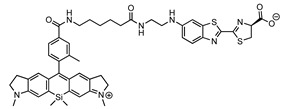 SiR700 X-AL	Fluc	712	[38,39]
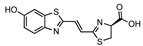 iLH2	Fluc Fluc S284T	670 706	[40,57]
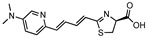 SeMpai	Fluc	675	[41,58,59]
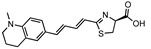 NIRLuc2	Fluc	683	[42]
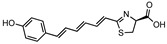 Hexatriene phenol luciferin	Flu	765	[43]
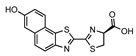 Hydroxy-naphtha[2,1] thiazole luciferin	CBR	740	[44]
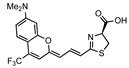 CoLuc-3-NMe_2_	Pistachio	710	[45]
